# Hirudin as an anticoagulant for both haematology and chemistry tests

**DOI:** 10.1155/S1463924600000158

**Published:** 2000

**Authors:** Takeo Kumura, Masayuki Hino, Takahisa Yamane, Noriyuki Tatsumi

**Affiliations:** 1 Departments of Laboratory Medicine Osaka City University Medical School Osaka Japan; 2 Departments of Clinical Hematology Osaka City University Medical School Osaka Japan

## Abstract

Hirudin, an extract from the leech, has powerful antithrombin activity affecting the blood coagulation pathway. We evaluated the usefulness of hirudin in anticoagulating specimens for routine laboratory tests. Results using blood anticoagulated with hirudin corresponded well with results with blood treated with ethylenediamine tetraacetic acid (EDTA) in the complete blood count (CBC), including white blood cell (WBC) differential count and morphology of blood cells, when CBC was performed within 2 h of blood collection. Clinical chemistry results from hirudin-treated samples were similar to results obtained with serum specimens. Thus, hirudin may be a useful anticoagulant for emergency laboratory medicine.


Journal of Automated Methods & Management in Chemistry, Vol. 22, No. 4 (July–August 2000) pp. 109–112

### Hirudin as an anticoagulant for both haematology and chemistry tests

**Takeo Kumura,[1] Masayuki Hino,[2] Takahisa Yamane[2] and Noriyuki Tatsumi[1,2]**

[1] *Departments of Laboratory Medicine and* [2] *Clinical Hematology, Osaka City University Medical School, Osaka, Japan*

*Hirudin, an extract from the leech, has powerful antithrombin activity affecting the blood coagulation pathway. We evaluated the usefulness of hirudin in anticoagulating specimens for routine laboratory tests. Results using blood anticoagulated with hirudin corresponded well with results with blood treated with ethylenediamine tetraacetic acid (EDTA) in the complete blood count (CBC), including white blood cell (WBC) differential count and morphology of blood cells, when CBC was performed within 2 h of blood collection. Clinical chemistry results from hirudin-treated samples were similar to results obtained with serum specimens. Thus, hirudin may be a useful anticoagulant for emergency laboratory medicine.*

### Introduction

Blood tests are used widely for clinical monitoring and diagnosis. Relatively high volumes of blood and multiple sampling tubes are usually required: 2 ml of blood in an EDTA-containing tube for haematology tests, 5 ml in an anticoagulant-free tube for most chemistry tests, and more blood in citrated tubes for others such as coagulation tests. These tubes of blood are loaded into different automated analysers and the entire sample is usually needed. Patients object to the frequency of phlebotomy for clinical tests. To decrease specimen waste, universal anticoagulants have been sought for clinical laboratory use.

EDTA is currently used for haematology tests requiring whole blood. A chelator of divalent cations, EDTA interferes with blood coagulation by removing calcium ions from the sample. While plasma obtained from whole blood containing EDTA is used for chemistry tests in some emergency laboratories, results of some chemistry tests are distorted both by chelation and by the sodium or potassium ions associated with EDTA. An alternative anticoagulant to EDTA is hirudin (figure 1), known to strongly inhibit blood coagulation by blocking the action of thrombin [1–4]. However, no trials have assessed its use as a laboratory anticoagulant. We studied the use of hirudin in specimens for haematology and chemistry tests.

### Materials and methods

### Reagents

After recombinant hirudin (American Diagnostica, Greenwich, CT, USA) was dissolved in distilled water (20 μg/0.5 ml), quantities of this solution were placed in blood specimen collection tubes to result in a final concentration of 4 μg hirudin/2.0 ml blood. Tubes containing 2.4 mg of dipotassium ethylenediamine tetraacetic acid (EDTA-2K) per 2 ml of blood (Terumo, Tokyo, Japan) were used as controls for haematology tests.

### Venous blood

Blood samples were collected from 30 healthy normal subjects (15 males and 15 females) and divided into hirudin-containing tubes for haematology and chemistry tests, EDTA-containing tubes for haematology control samples, and anticoagulant-free tubes as serum controls for chemistry tests. Tubes for haematology tests were allowed to stand for at least 15 min at room temperature to stabilize samples before testing. The serum was separated by centrifugation at 400 ***g*** for 5 min after leaving for 60 min. Hirudin-treated plasma was separated by centrifugation at 400 ***g*** for 5 min. All tests were completed within 2 h of blood collection.

### Haematology studies

CBC and WBC differential counts were performed with a haematology analyser (NE-8000, Toa Medical Electronics, Kobe, Japan). Parameters evaluated were white blood cell count (WBC), red blood cell count (RBC), haemoglobin (Hb), haematocrit (Hct), mean corpuscular volume (MCV), mean corpuscular haemoglobin (MCH), mean corpuscular haemoglobin concentration (MCHC), platelet count (Plt), and percentages of the WBC representing neutrophils, lymphocytes, monocytes, eosinophils and basophils.

### Morphology of blood cells

Blood samples taken from tubes within 2 h after collection were smeared on slides, fixed and stained by the Giemsa method for microscopic observation.

### Stability of haematology specimens

CBC and WBC differential counts were compared over time immediately and at 1, 2 and 3 h after blood collection using the NE-8000 analyser at 25 °C for samples anticoagulated with either EDTA or hirudin.

### Reproducibility of haematology parameters

Samples anticoagulated with either EDTA or hirudin from healthy adults ($n = 5$) were assayed five times, and the coefficients of variation (CV) for CBC and WBC differential counts were calculated.

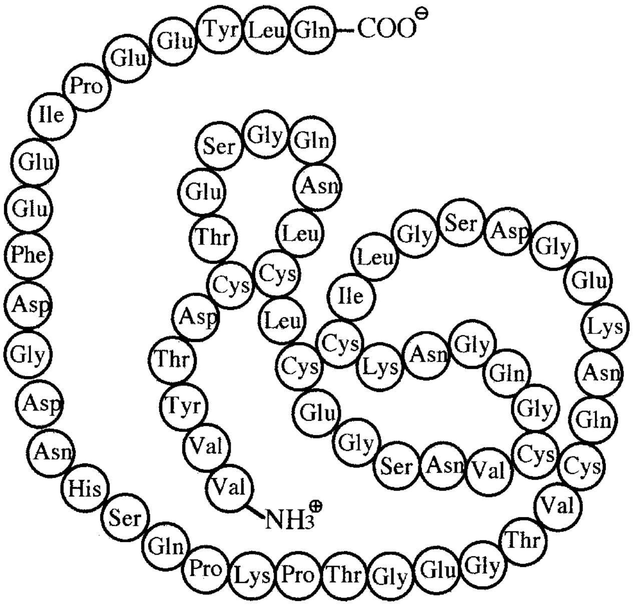

*Figure 1. Structural formula of recombinant hirudin.*

### *Chemistry tests*

Chemistry tests were performed using a fully automated analyser (7170 Automatic Analyzer, Hitachi, Tokyo, Japan) for determinations of aspartate aminotransferase (AST, IU/l), alanine aminotransferase (ALT, IU/l), total bilirubin (T-Bil, mg/dl), lactate dehydrogenase (LDH, IU/l), blood urea nitrogen (BUN, mg/dl), creatinine (Cre, mg/dl), sodium (Na, mEq/l), potassium (K, mEq/l), chloride (Cl, mEq/l), total protein (TP, g/dl), albumin (Alb, g/dl), total cholesterol (T-Cho, mg/dl), triglyceride (TG, mg/dl), alkaline phosphatase (ALP, IU/l), leucine aminopeptidase (LAP, IU/l), cholinesterase (Ch-E, IU/l), gamma glutamyl transpeptidase ($\gamma$-GTP, IU/l) and uric acid (UA, mg/dl).

### *Statistics*

Statistical analysis was carried out using the Stat View-4.5J software package (Abacus Concepts, Berkeley, CA, USA). A paired $t$-test was used for comparison analyses; $P$ values less than 0.01 were considered significant.

### Results

### *Haematology tests*

No significant differences were noted for any item between EDTA-treated and hirudin-treated blood (table 1). Any possible effects of hirudin on blood cell morphology were meticulously sought. No effects were seen on white cells, red cells or platelet counts.

### *Stability of haematology specimens*

For 2 h after blood collection, no significant difference was seen in CBC or differential WBC counts between EDTA-treated blood and hirudin-treated blood. However, when the measurement was performed 3 h after collection, WBC, RBC and platelet counts showed decreases due to coagulation in hirudin-treated blood (figure 2). Four hours later, the specimen had completely coagulated and measurements could not be performed.

*Table 1. CBC parameters and WBC differential count with EDTA and hirudin anticoagulation at 2h after blood collection.*

| *Item* | *EDTA* | *Hirudin* | *P** |
|---|---|---|---|
| WBC ($\times 10^2/\mu l$) | 52.3 ± 7.5 | 51.6 ± 7.4 | NS |
| RBC ($\times 10^4/\mu l$) | 415.2 ± 49.3 | 418.1 ± 41.5 | NS |
| Hb (g/dl) | 13.3 ± 1.6 | 13.1 ± 1.5 | NS |
| Hct (%) | 36.5 ± 4.2 | 35.9 ± 3.9 | NS |
| MCV (fl) | 90.2 ± 6.4 | 89.4 ± 6.7 | NS |
| MCH (pg) | 30.9 ± 0.8 | 30.8 ± 0.9 | NS |
| MCHC (g/dl) | 34.3 ± 1.1 | 34.5 ± 1.3 | NS |
| Plt ($\times 10^4/\mu l$) | 29.7 ± 4.2 | 29.1 ± 4.1 | NS |
| Neutrophils (%) | 62.2 ± 3.6 | 61.5 ± 3.5 | NS |
| Lymphocytes (%) | 29.5 ± 10.8 | 29.8 ± 10.5 | NS |
| Basophils (%) | 0.6 ± 0.3 | 0.6 ± 0.4 | NS |
| Eosinophils (%) | 2.9 ± 1.8 | 3.2 ± 1.7 | NS |
| Monocytes (%) | 4.8 ± 1.8 | 4.9 ± 1.8 | NS |

Values are mean ± SD. * By paired $t$-test. NS, not significant. $n = 30$.

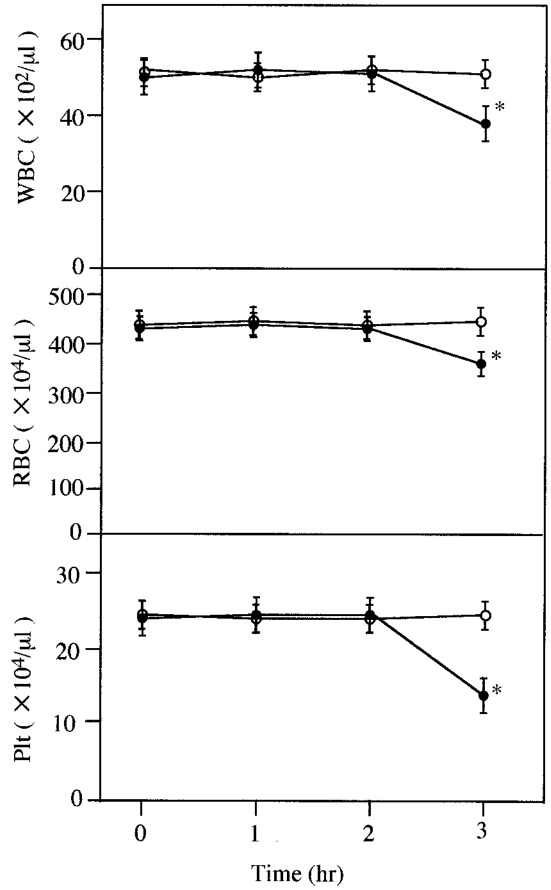

*Figure 2. Serial changes of WBC, RBC and platelet count (Plt) with EDTA and hirudin anticoagulation. —○—, EDTA-treated blood; —●—, hirudin-treated blood. Data are mean± SD ($n = 30$). *$P < 0.01$ versus corresponding EDTA-treated blood by paired t-test.*

*Table 2. Coefficient of variation (CV) for CBC parameters and WBC differential count with EDTA and hirudin anticoagulation.*

| Item | CV(%) | | |
|---|---|---|---|
| | EDTA | Hirudin | P* |
| WBC | 1.86 ± 0.25 | 1.98 ± 0.36 | NS |
| RBC | 1.69 ± 0.43 | 1.64 ± 0.46 | NS |
| Hb | 1.02 ± 0.38 | 1.09 ± 0.41 | NS |
| Fct | 1.08 ± 0.29 | 1.12 ± 0.31 | NS |
| MCV | 0.45 ± 0.21 | 0.41 ± 0.18 | NS |
| MCH | 0.81 ± 0.19 | 0.75 ± 0.18 | NS |
| MCHC | 0.45 ± 0.18 | 0.42 ± 0.15 | NS |
| Plt | 4.5 ± 1.18 | 4.7 ± 1.21 | NS |
| Neutrophils | 2.75 ± 1.03 | 2.88 ± 1.08 | NS |
| Lymphocytes | 4.44 ± 1.31 | 4.51 ± 1.22 | NS |
| Basophils | 18.1 ± 9.9 | 18.6 ± 10.1 | NS |
| Eosinophils | 15.9 ± 10.2 | 16.6 ± 10.9 | NS |
| Monocytes | 13.3 ± 4.49 | 12.6 ± 4.52 | NS |

Values are mean ± SD. * By paired *t*-test. NS, not significant. $n = 5$.

*Reproducibility of haematology parameters*

Good reproducibility was demonstrated for each item and no significant differences were noted between EDTA-treated and hirudin-treated blood (table 2). The results indicated that the reliability of determinations performed by the haematology analyser was not affected by hirudin.

*Chemistry tests*

Significant differences were seen for potassium ($P < 0.01$) and TP ($P < 0.01$) between hirudin-treated plasma and serum (table 3). However, very good correlations were noted between hirudin-treated plasma and serum for potassium ($r = 0.925$) and TP ($r = 0.974$) (figure 3).

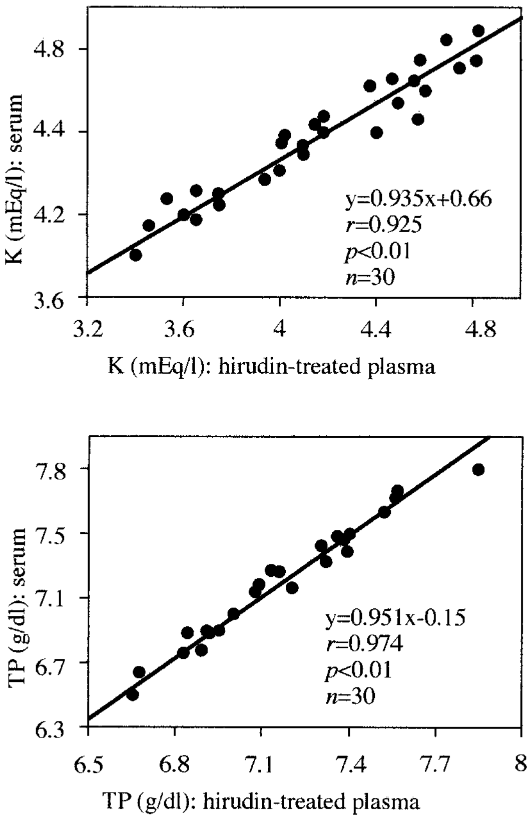

*Figure 3. Correlations for potassium (K) and total protein (TP) concentrations between serum and hirudin-treated plasma.*

*Table 3. Commonly measured chemical components in serum and hirudin-treated plasma.*

| Item | Serum | Hirudin-treated plasma | P* |
|---|---|---|---|
| AST (IU/l) | 20 ± 8 | 21 ± 7 | NS |
| ALT (IU/l) | 17 ± 6 | 16 ± 5 | NS |
| T-Bil (mg/dl) | 0.5 ± 0.1 | 0.5 ± 0.1 | NS |
| LDH (IU/l) | 331 ± 74 | 334 ± 52 | NS |
| BUN (mg/dl) | 13.9 ± 4.7 | 13.7 ± 4.7 | NS |
| Cre (mg/dl) | 0.8 ± 0.1 | 0.8 ± 0.1 | NS |
| Na (mEq/l) | 139 ± 4 | 140 ± 4 | NS |
| K (mEq/l) | 4.5 ± 0.4 | 4.1 ± 0.3 | <0.01 |
| Cl (mEq/l) | 103 ± 3 | 104 ± 3 | NS |
| TP (g/dl) | 6.6 ± 0.5 | 7.1 ± 0.5 | <0.01 |
| Alb (g/dl) | 3.6 ± 0.4 | 3.6 ± 0.4 | NS |
| T-Cho (mg/dl) | 159 ± 33 | 154 ± 32 | NS |
| TG (mg/dl) | 81 ± 23 | 82 ± 24 | NS |
| ALP (IU/l) | 169 ± 48 | 167 ± 49 | NS |
| LAP (IU/l) | 97 ± 21 | 96 ± 22 | NS |
| Ch-E (IU/l) | 158 ± 53 | 156 ± 52 | NS |
| γ-GTP (IU/l) | 24 ± 11 | 23 ± 10 | NS |
| UA (g/dl) | 3.9 ± 1.1 | 3.8 ± 1.1 | NS |

Values are mean ± SD. * By paired *t*-test. NS, not significant. $n = 30$.

### Discussion

Most physicians order multiple laboratory tests to manage patients, thereby requiring substantial volumes of blood. Tubes for haematology tests have 2- or 5-ml capacities, while tubes for chemistry tests have 5–10-ml capacities, exceeding volumes required for the tests. A single collection tube for all tests would be desirable. Hirudin was studied in this context as an anticoagulant for haematology and chemistry tests. Recombinant hirudin was used in our study as an anticoagulant for blood collection. Hirudins, a group of highly homologous polypeptides present in the medicinal leech (*Hirudo medicinalis*), have an extremely high affinity for thrombin, and are consequently potent anticoagulants [5, 6]. Recombinant hirudins have been produced from either synthetic genes or from cDNA isolated from leeches. In clinical trials the recombinant hirudins have been confirmed to be highly selective and potent inhibitors of thrombin [7–10].

EDTA, a potent calcium ion chelator, is used widely for haematology tests because it assures stable measurements for at least 12 h after blood collection. Stability and reliability of CBC and automated WBC differential count results using EDTA has worked against development of new reagents. Nevertheless, our results showed that hirudin-treated and EDTA-treated blood gave similar values for CBC and WBC differential counts, and no morphologic changes due to hirudin could be observed within 2 h of blood collection. While hirudin inhibits thrombin selectively, clotting can eventually get underway because of the incredible rapidity with which thrombin is generated, faster than the association rate of hirudin unless very high concentrations are used [11].

EDTA-treated plasma is used for chemistry tests in some emergency laboratories. Thus, as it usually takes 40–50 min to obtain a serum specimen for chemistry tests, importance was attached to the experiment with plasma. However, measurements of calcium, iron, alkaline phosphatase, leucine aminopeptidase, and sodium and potassium are distorted by chelation and by sodium or potassium ions associated with EDTA. Correspondence between results using serum and hirudin-treated plasma was found to be high. Significant differences between hirudin-treated plasma and serum were seen for potassium $(P < 0.01)$ and TP $(P < 0.01)$ concentrations. The difference for potassium may have resulted from release of platelet-derived potassium in serum during blood coagulation which does not occur in hirudin-treated plasma. The TP value was higher in hirudin-treated plasma than in serum because hirudin-treated plasma contains fibrinogen. Because differences between such plasma and serum were limited and predictable, hirudin may be useful for emergency laboratory use as well as for maximizing diagnostic yield from small blood samples.

The number of samples evaluated in the present small pilot study was not large, and the ranges of values compared were limited, but our data indicate suitability of hirudin anticoagulation for haematology and chemistry test specimens. While these data suggest that hirudin is an attractive candidate as an anticoagulant for haematology and chemistry tests, further laboratory trials for coagulation tests are necessary.

### References

1. Wallis, R. B., 1996, *Seminars in Thrombosis and Hemostasis*, **22**, 185.
2. Dodt, J., Muller, H. P., Seemuller, U. and Chang, J. U., 1984, *Federation of European and Biochemical Societies*, **165**, 180.
3. Markwardt, F., 1989, *Seminars in Thrombosis and Hemostasis*, **15**, 269.
4. Maraganore, J. M., Bourdon, O., Jablonski, J., Ramachandran, K. L. and Fenton, J. W., 1990, *Biochemistry* , **29**, 7095.
5. Rydel, T. J., Ravichandran, K. G., Tukinsky, A., Bode, W., Huber, R., Roitsch, C. and Fenton, J. W., 1990, *Science*, **249**, 277.
6. Haruyama, H. and Wuthrich, K., 1990, *Biochemistry* , **28**, 4301.
7. Talbot, M., 1989, *Seminars in Thrombosis and Hemostasis*, **15**, 293.
8. Marki, W. E., Grossenbacher, H., Grutter, M. G., Liersch, M. H., Meyhack, B. and Heim, J., 1991, *Seminars in Thrombosis and Hemostasis*, **17**, 88.
9. Braun, P. J., Dennis, S., Hofsteenge, J. and Stone, S. R., 1988, *Biochemistry* , **27**, 1517.
10. Agnelli, G., Renga, C., Weitz, J. I., Nenci, G. G. and Hirsh, J., 1992, *Blood*, **80**, 960.
11. Lindhout, T., Blezer, R. and Hemker, H. C., 1990, *Thrombosis and Haemostasis*, **64**, 454.

